# Eating speed and height loss in relation to overweight: A retrospective study

**DOI:** 10.1371/journal.pone.0284998

**Published:** 2023-04-26

**Authors:** Yuji Shimizu, Hidenobu Hayakawa, Eiko Honda, Nagisa Sasaki, Midori Takada, Takeo Okada, Testuya Ohira, Masahiko Kiyama

**Affiliations:** 1 Epidemiology Section, Division of Public Health, Osaka Institute of Public Health, Osaka, Japan; 2 Department of Cardiovascular Disease Prevention, Osaka Center for Cancer and Cardiovascular Diseases Prevention, Osaka, Japan; 3 Department of Epidemiology, Fukushima Medical University School of Medicine, Fukushima, Japan; University of Minnesota School of Dentistry, UNITED STATES

## Abstract

Fast eating is an independent risk factor for weight gain. Our previous study involving Japanese workers revealed that overweight (body mass index ≥ 25.0 kg/m^2^) is an independent risk factor for height loss. However, no studies have clarified the association between eating speed and height loss in relation to overweight status. A retrospective study of 8,982 Japanese workers was conducted. Height loss was defined as being in the highest quintile of height decrease per year. Compared with slow eating, fast eating was revealed to be positively associated with overweight; the fully adjusted odds ratio (OR) and 95% confidence interval (CI) was 2.92 (2.29, 3.72). Among non-overweight participants, fast eaters had higher odds of height loss than slow eaters. Among overweight participants, fast eaters had lower odds of height loss; the fully adjusted OR (95% CI) was 1.34 (1.05, 1.71) for non-overweight individuals and 0.52 (0.33, 0.82) for overweight individuals. Since overweight was significantly positively associated with height loss [1.17(1.03, 1.32)], fast eating is not favorable for reducing the risk of height loss among overweight individuals. Those associations indicate that weight gain is not the main cause of height loss among Japanese workers who eat fast.

## 1. Introduction

Recently, eating speed was revealed to be associated with cardiovascular risk factors. Fast eating might increase the risk of cardiovascular disease by increasing the incidence of the components of metabolic syndrome [1, 2]. Another study reported that height loss starting in middle age is an independent risk factor for cardiovascular mortality [3].

Since both eating speed and height loss are associated with cardiovascular risk factors, faster eating speed could be positively associated with height loss. However, there have been no reported studies about the association between eating speed and height loss.

In addition, fast eating is significantly positively associated with overweight or obesity [4]. Overweight is an independent risk for height loss among Japanese workers [5]. Therefore, overweight could strongly influence the association between eating speed and height loss.

Previously, circulating CD34-positive cell count was revealed to be inversely associated with height loss [6]. Circulating CD34-positive cells play a major role in endothelial repair [7, 8]. Since height loss is an independent risk factor for cardiovascular mortality [3] and circulating CD34-positive cell count is reported to be inversely associated with cardiovascular mortality [9], endothelial repair activity might affect the associations among eating speed, height loss, overweight, and cardiovascular disease.

Because intervertebral disc degeneration and vertebral fractures play important roles in height loss among adults, clarifying the risk factors for height loss might also help efficiently identify novel mechanisms contributing to intervertebral disc degeneration and vertebral fractures.

In addition, overweight is reported to be associated with structural atherosclerosis as evaluated by carotid intima-media thickness [10]. The development of structural atherosclerosis requires circulating CD34-positive cells [11]. Since a shortage of circulating CD34-positive cells is a significant risk factor for height loss [6], overweight could act as a strong confounder on the association between eating speed and height loss. Being overweight might stimulate the production of CD34-positive cells.

Therefore, we hypothesized the following. First, at baseline, independent of known cardiovascular risk factors, the prevalence of overweight is significantly higher among fast eaters than among slow eaters. Second, overweight at baseline is an independent risk factor for height loss. We hypothesized that, independent from known cardiovascular risk factors, eating speed is significantly positively associated with height loss only among participants who are not overweight because overweight might act as a strong confounder on the association between eating speed and height loss.

To test our hypotheses, we conducted a cross-sectional study and a retrospective study of Japanese workers.

## 2. Materials and methods

The detail of this survey has been described elsewhere [5].

### 2.1. Study population

Since 2008, the Ministry of Health, Labour and Welfare of Japan has been promoting a medical examination specifically for cardiovascular disease prevention.

The present study was performed at the Osaka Center for Cancer and Cardiovascular Diseases Prevention. The study population comprised 9,681 general workers aged 40–74 years who underwent the specific medical examinations between 2011 and 2017 (baseline). Two participants without data on eating speed were excluded from the study population. Participants of the present study were individuals who were able to engage in work. Therefore, the participants might have been relatively healthier than the general population. In addition, men are more likely to work than women in Japanese society. Thus, the proportion of men might be higher than in the general population.

Consent for this study was obtained using the opt-out method. Descriptions of the study were posted on posters and the institution’s website (www.osaka-ganjun.jp/effort/cvd/r-and-d/). The ethics committee of the Osaka Center for Cancer and Cardiovascular Diseases Prevention approved the present study (project registration code, R4-Rinri-4).

To calculate height decrease per year for the present study, at least two height measurements (baseline and endpoint) during the observation period were necessary. Thus, participants without height measured during 2012–2018 (endpoint) were excluded from the analysis (n = 697). Therefore, 8,982 participants were included in this retrospective study. The mean age [standard deviation (SD)] of study participants was 50.6 (8.3) years.

Mean follow-up was 3.5 years (SD, 2.0 years; interquartile range, 1.9–5.7 years).

### 2.2. Data collection and laboratory measurements

#### 2.2.1. Baseline data

In this study, data from participants who underwent annual health examinations during 2011 and 2017 were used as baseline data. Data on medication history and drinking status (none, often, daily) and smoking status (never, former, current smoker) were acquired by trained interviewers. Eating speed was self-reported according to one of three categories: slow, moderate, and fast.

To calculate body mass index (BMI), height in feet while wearing stockings and weight while wearing light clothing were measured. Trained nurses collected fasting blood samples, which were used to measure hemoglobin, total cholesterol (TC), triglycerides (TG), high-density lipoprotein cholesterol (HDLc), hemoglobin A1c (HbA1c), and serum creatinine using standard procedures at the Osaka Center for Cancer and Cardiovascular Diseases Prevention. The Friedewald formula was used to calculate low-density lipoprotein cholesterol (LDLc) concentration: LDLc (mg/dL) = TC-(TG/5)-HDLc.

Before 2012, HbA1c values were measured throughout Japan using the Japanese Diabetes Society (JDS) definition. Since 2013, the National Glycohemoglobin Standardization Program (NGSP) definition was used. Upon this change, the JDS working group proposed the following equation to convert values: HbA1c (NGSP) = HbA1c (JDS) + 0.4% [12]. We used this equation for HbA1c data measured during 2011 and 2012. According to World Health Organization guidelines for Asians [13], overweight was defined as BMI ≥ 25 kg/m^2^. Hypertension was defined as systolic blood pressure ≥ 140 mmHg, diastolic blood pressure ≥ 90 mmHg, or use of anti-hypertensive medication. Dyslipidemia was defined as TG ≥ 150 mg/dL, LDLc ≥ 140 mg/dL, HDLc < 40 mg/dL, or use of lipid-lowering medication. Diabetes was defined as HbA1c (NGSP) ≥ 6.5% or use of glucose-lowering medication.

#### 2.2.2. Endpoint data

Height and weight were measured at the endpoint. BMI and height change per year were calculated. Height loss was defined as being the highest quintile of annual height decrease, as in our previous studies (1.78 mm/year for men and 2.06 mm/year for women) [5, 14].

### 2.3. Statistical analysis

Clinical characteristics related to overweight status were expressed as means ± SD for continuous variables such as age at baseline and BMI at the endpoint. They were also stratified by eating speed. The prevalence of men, drinking, smoking, hypertension, diabetes, and dyslipidemia by eating speed and stratified by overweight status was also calculated. Significant differences were evaluated using analysis of variance (ANOVA) for continuous variables and the chi-squared test for proportions.

Logistic regression was used to evaluate the association between eating speed and overweight and between overweight and the incidence of height loss. We also used logistic regression to calculate odds ratios (ORs) and 95% confidence intervals (CIs) for height loss with respect to eating speed, stratified by overweight status.

A mechanism that is cardiovascular in nature might be underlying the association between eating speed and height loss [1–3]. Three different approaches were used to make adjustment for confounding factors. Model 1 adjusted only for sex and age. Model 2 included several other potential confounding factors in addition to sex and age, namely drinking status (none, often, daily), smoking status (never, former, current), hypertension (yes, no), dyslipidemia (yes, no), and diabetes (yes, no). For the analysis of eating speed and height loss, we included the variables in Model 2 plus BMI at the endpoint as a confounder (Model 3).

For sensitivity analysis, sex-specific analysis and age group-stratified analysis (40 to 49 years, 50 to 59 years, and 60 to 74 years) were performed similar to the main analysis. We also re-ran the main analysis with height loss defined as being in the highest sextile of annual height decrease.

Values of p<0.05 were regarded as statistically significant. All statistical analyses were performed with SAS for Windows (version 9.4; SAS Inc., Cary, NC, USA).

## 3. Results

### 3.1. Characteristics of the study population

As shown in Table 1, in both non-overweight and overweight study participants, eating speed was positively associated with male sex and inversely associated with age. Other values shown in Table 1 are sex- and age-adjusted values (least squares mean) for the study population by eating speed. Among participants who were not overweight, often drinker status and former smoker status were each positively associated with eating speed. Among overweight participants, daily drinker status and current smoker status were each inversely associated with eating speed.

**Table 1:** Characteristics of the study population by eating speed and overweight status.

	Non-overweight (BMI < 25.0 kg/m^2^)				Overweight (BMI ≥ 25.0 kg/m^2^)			
	Eating speed			p for trend	Eating speed			p for trend
	Slow	Moderate	Fast		Slow	Moderate	Fast	
No. of participants	612	3680	2368		93	1091	1138	
Men, %	54.9	53.3	58.4	<0.001	73.1	77.4	81.2	0.030
Age, years	50.8 ± 8.8	50.7 ± 8.4	50.2 ± 8.1	0.039	52.7 ± 9.4	51.4 ±8.3	50.5 ± 8.1	0.004
Daily drinker, %	21.2	21.5	22.0	0.380	25.8	18.9	15.9	0.021
Often drinker, %	38.9	43.1	46.0	0.003	41.9	50.2	51.4	0.209
Current smoker, %	26.1	25.1	24.4	0.629	33.3	30.8	27.9	0.239
Former smoker, %	25.7	27.4	30.6	0.009	28.0	35.1	38.2	0.071
Hypertension, %	18.1	17.9	19.1	0.497	41.9	40.4	41.0	0.932
Diabetes, %	4.6	4.3	3.7	0.454	12.9	13.1	12.0	0.746
Dyslipidemia, %	35.9	36.5	38.0	0.459	57.0	63.2	66.5	0.079
BMI at the endpoint, kg/m^2^	20.9 ± 2.5	21.5 ± 2.3	21.9 ± 2.2	<0.001	27.2 ± 3.1	27.5 ± 2.8	27.8 ± 3.3	0.020

Age and BMI at the endpoint are shown as means ± standard deviation. Other variables are shown as percentages. BMI: body mass index.

### 3.2. Association between eating speed and overweight

Independent of known confounding factors, eating speed was significantly positively associated with overweight (Table 2). With slow eaters as the reference group, the fully adjusted OR (95% CI) for overweight was 1.96 (1.54, 2.50) for moderate-speed eaters and 2.92 (2.29, 3.72) for fast eaters.

**Table 2:** Association between eating speed and overweight status.

	Eating speed			p for trend
	Slow	Moderate	Fast	
No. of participants	705	4771	3506	
No. of overweight (%)	93 (13.2)	1091 (22.9)	1138 (32.5)	
Model 1	Reference	1.97 (1.56, 2.48)	3.05 (2.42, 3.85)	<0.001
Model 2	Reference	1.96 (1.54, 2.50)	2.92 (2.29, 3.72)	<0.001

Model 1: adjusted only for sex and age. Model 2: adjusted for sex, age, drinking status (none, often, daily), smoking status (never, former, current), hypertension, dyslipidemia, and diabetes.

### 3.3. Association between overweight and height loss

As shown in Table 3, overweight was positively associated with height loss. The sex- and age-adjusted OR (95% CI) for height loss and overweight was 1.21 (1.07, 1.36). This association was unchanged even after further adjusting for known confounding factors. The fully adjusted OR (95% CI) was 1.17 (1.03, 1.32).

**Table 3:** Association between overweight status and height loss.

	Non-overweight	Overweight	p
No. of participants	6660	2322	
No. with height loss (%)	1281 (19.2)	515 (22.2)	
Model 1	Reference	1.21 (1.07, 1.36)	0.002
Model 2	Reference	1.17 (1.03, 1.32)	0.016

Model 1: adjusted only for sex and age. Model 2: adjusted for sex, age, drinking status (none, often, daily), smoking status (never, former, current), hypertension, dyslipidemia, and diabetes.

### 3.4. Association between eating speed and height loss by overweight status

Table 4 shows the ORs (95% CIs) for height loss and eating speed stratified by overweight status. Among non-overweight participants, independent of known confounding factors including BMI at the endpoint, eating speed was significantly positively associated with height loss. With slow eaters as the reference group, the fully adjusted OR (95% CI) for height loss was 1.26 (1.00, 1.59) for moderate-speed eaters and 1.34 (1.05, 1.71) for fast eaters. Among overweight participants, eating speed was significantly inversely associated with height loss independent of known confounding factors including weight change per year. With slow eaters as the reference group, the fully adjusted OR (95% CI) for height loss was 0.60 (0.38, 0.94) for moderate-speed eaters and 0.52 (0.33, 0.82) for fast eaters.

**Table 4:** Association between eating speed and height loss by overweight status.

			Eating speed			p for trend
			Slow	Moderate	Fast	
**Non overweight**						
	No. of participants		612	3680	2368	
	No. with height loss (%)		99 (16.2)	711 (19.3)	471 (19.9)	
	Model 1		Reference	1.25 (0.99, 1.58)	1.33 (1.05, 1.69)	0.037
	Model 2		Reference	1.25 (0.99, 1.58)	1.33 (1.05, 1.69)	0.036
	Model 3		Reference	1.26 (1.00, 1.59)	1.34 (1.05, 1.71)	0.030
**Overweight**						
	No. of participants		93	1091	1138	
	No. with height loss (%)		32 (34.4)	253 (23.2)	230 (20.2)	
	Model 1		Reference	0.60 (0.38, 0.95)	0.52 (0.33, 0.83)	0.013
	Model 2		Reference	0.60 (0.38, 0.94)	0.52 (0.53, 0.82)	0.012
		Model 3	Reference	0.60 (0.38, 0.94)	0.52 (0.33, 0.82)	0.012

Model 1: adjusted only for sex and age. Model 2: adjusted for sex, age, drinking status (none, often, daily), smoking status (never, former, current), hypertension, dyslipidemia, and diabetes. Model 3: adjusted the variables in Model 2 and further adjusted for body mass index (BMI) at the endpoint.

### 3.5. Sex-specific analyses

Essentially the same associations were observed for men and women. Compared with slow eaters, the age-adjusted sex-specific OR (95% CI) for overweight was 2.12 (1.62, 2.79) for male moderate-speed eaters and 3.29 (2.50, 4.33) for female moderate-speed eaters; for fast eaters, they were 1.57 (1.02, 2.42) and 2.38 (1.54, 3.68), respectively.

Positive associations between height loss and overweight were observed for both men and women. The age-adjusted OR (95% CI) for height loss and overweight was 1.16 (1.01, 1.34) for men and 1.31 (1.04, 1.65) for women.

Among non-overweight participants, there were non-statistically significant positive associations between eating speed and height loss in men and women. With slow eaters as the reference group, the age-adjusted OR (95% CI) for height loss was 1.27 (0.93, 1.72) for male moderate-speed eaters and 1.22 (0.86, 1.73) for female moderate-speed eaters; they were 1.24 (0.90, 1.70) and 1.41 (0.98, 2.03) for male and female fast eaters, respectively.

Among overweight participants, essentially the same inverse association between eating speed and height loss was observed. With slow eaters as the reference group, the age-adjusted OR (95% CI) for height loss was 0.59 (0.35, 1.01) for male moderate-speed eaters and 0.62 (0.26, 1.50) for female moderate-speed eaters; they were 0.50 (0.29, 0.85) and 0.60 (0.24, 1.45) for male and female fast eaters, respectively.

### 3.6. Associations between eating speed and height loss stratified by age group

To evaluate the influence of age group on the association between eating speed and height loss by overweight status, age group-stratified analysis was also performed. The associations were essentially the same. Among non-overweight participants aged 40 to 49 years (n = 3,409), when compared with slow eaters, the sex- and age-adjusted ORs (95% CI) for height loss were 1.08 (0.77, 1.51) for moderate-speed eaters and 1.23 (0.87, 1.73) for fast eaters, respectively. The corresponding values were 1.41 (0.90, 2.21) and 1.50 (0.95, 2.58) for participants aged 50 to 59 years (n = 2,065) and 1.44 (0.91, 2.27) and 1.32 (0.81, 2.14) for participants aged 60 to 74 years (n = 1,186).

Among overweight participants, the corresponding values were 0.63 (0.30, 1.30) and 0.68 (0.33, 1.40) for participants aged 40 to 49 years (n = 1,107), 0.40 (0.18, 0.90) and 0.27 (0.12, 0.60) for participants aged 50 to 59 years (n = 785), and 0.87 (0.37, 2.09) and 0.77 (0.32, 1.86) for participants aged 60 to 74 years (n = 430).

### 3.7. Associations between height loss defined as being in the highest sextile of annual height decrease

In addition, we performed the same analyses with height loss defined as being in the highest sextile of annual height decrease. The association between height loss and overweight status and the associations between height loss and eating speed stratified by overweight status were also calculated. The sex- and age-adjusted OR (95% CI) for height loss with respect to overweight was 1.16 (1.02, 1.31). Among non-overweight participants, compared with slow eaters, the sex- and age-adjusted ORs (95% CI) for height loss were 1.31 (1.02, 1.69) for moderate-speed eaters and 1.33 (1.02, 1.72) for fast eaters, respectively. Among overweight participants, the corresponding values were 0.67 (0.41, 1.10) and 0.57 (0.36, 0.96), respectively.

## 4. Discussion

The major finding of the present study is that eating speed was significantly positively associated with height loss among participants who were not overweight but significantly inversely associated with height loss among those who were overweight.

In the present study, additional analyses showed essentially the same associations between men and women. Therefore, sex might not have affected the main results. In addition, another analysis stratified by age group (40 to 49 years, 50 to 59 years, and 60 to 74 years) showed essentially the same associations. Thus, age group also might not have affected the main results.

A systematic review reported that non-fast eaters had significantly lower BMI than fast eaters [15]. In the present study, we found a significant positive association between eating speed and overweight.

Although eating speed is significantly and positively correlated with total energy intake, independent of total energy intake and other confounders, eating speed is positively associated with overweight [16]. A previous study with middle-aged Japanese men and women reported an independent association between insulin resistance and eating speed [17]. Since insulin resistance is positively associated with high BMI [18], factors related to insulin resistance might also contribute to weight gain in fast eaters independent of total energy intake.

In addition, our previous study with 6,471 men and 3,180 women aged 40 to 74 years revealed that overweight (BMI ≥ 25 kg/m^2^) is independently positively associated with height loss when defined as being in the highest quintile of annual height loss. The fully adjusted OR (95% CI) for height loss and overweight was 1.29 (1.13, 1.47) for men and 1.36 (1.06, 1.74) for women, respectively [5]. Lumbar intervertebral disc degeneration is the most frequent cause of height loss among the general adult population. Participants with disc degeneration have been reported to have higher BMI than participants without disc degeneration [19]. Furthermore, obesity is positively associated with intervertebral disc disorder [20]. Therefore, disc degeneration might play an important role in the relationship between overweight and height loss among the general adult population.

Therefore, the positive association between eating speed and height loss observed among participants who were not overweight (BMI < 25 kg/m^2^) could be partly explained by weight gain during the observation period, which is related to disc degeneration. However, even after adjusting for BMI at the endpoint, eating speed was significantly positively associated with height loss. Furthermore, among non-overweight and overweight patients, eating speed was significantly positively associated with BMI at the endpoint. However, the opposite associations between eating speed and height loss were observed; among non-overweight participants, eating speed was significantly positively associated with height loss whereas among overweight participants, eating speed was significantly inversely associated with height loss. Thus, weight gain during the observation period might not be the main explanation for the association observed among participants who were not overweight.

A previous study with 30 healthy subjects who did not use any medications found that the increase in serum glucose levels was higher and steeper with fast ingestion of sugary beverages (apple juice) than with slow ingestion [21]. Therefore, fast eaters might have a higher risk of post-prandial hyperglycemic spikes than slow eaters. Since post-prandial hyperglycemic spikes might be relevant to the development of cardiovascular disease [22] and height loss starting in middle age is reported to be an independent risk factor for cardiovascular mortality [3], post-prandial hyperglycemic spikes might be underlying the positive association between eating speed and height loss among participants who were not overweight. Furthermore, a previous study with healthy individuals free from diabetes reported a significant positive association between post-challenge hyperglycemic spikes and functional arterial stiffness evaluated with the cardio-ankle vascular index (CAVI) [23]. Since insufficient endothelial repair due to a shortage of hematopoietic stem cells known as CD34-positive cells accelerates functional atherosclerosis as evaluated by CAVI [7] and a shortage of CD34-positive cells is an independent risk for height loss among men aged 60 to 69 years [6], increased functional arterial stiffness due to post-prandial hyperglycemic spikes might explain the association between eating speed and height loss among non-overweight individuals. Since hypoxia accelerates intervertebral disc degeneration [24, 25], inadequate endothelial repair related to lower adaptability to hypoxia might play an important role in the development of intervertebral disc degeneration. Because lower adaptability to hypoxia is positively associated with functional atherosclerosis [8] and intervertebral disc degeneration is the major cause of height loss among adults, eating speed and height loss could be associated with functional atherosclerosis. Therefore, eating speed could be positively associated with height loss, which serves as an indicator of insufficient endothelial repair.

However, we also found a significant inverse association between eating speed and height loss among participants who were overweight. Overweight itself is an independent risk factor for height loss [5] and faster eaters gain weight [15]. Thus, among participants who were overweight, weight gain related to eating speed might not increase the risk of height loss. Compared to slow eaters, fast eaters might have a higher risk of post-prandial hyperglycemic spikes, which are related to the development of cardiovascular disease [21, 22]. However, overweight is also known to increase cardiovascular mortality [13]. Overweight is an independent risk factor for height loss [5] and height loss is reported to be associated with cardiovascular disease [3]. Therefore, the influence of post-prandial hyperglycemic spikes on height loss could be masked by overweight status.

In the present study, eating speed was significantly inversely associated with daily drinking among participants who were overweight. In addition, there was an inverse tendency between eating speed and current smoker status observed among participants who were overweight. However, this inverse tendency was not significant.

Heavy alcohol consumption reduces bone density, which is a known cause of osteoporosis [26]. Furthermore, the negative impact of obesity and smoking on the occurrence of lumbar disc herniation has been reported [27]. Since intervertebral disc disorder and vertebral fractures related to osteoporosis are well-known causes of height loss in adults, alcohol consumption and smoking might have influenced the association between eating speed and height loss among participants who were overweight. To clarify those mechanisms, further investigations with a larger number of participants who are overweight that include detailed information about alcohol consumption and smoking status are necessary.

Previously, LDLc was revealed to be positively associated with structural atherosclerosis and inversely associated with functional atherosclerosis [28], possibly by stimulating the proliferation of CD34-positive cells [29]. This previous study [28] showed that the development of structural atherosclerosis, a process of aggressive endothelial repair [8, 11] might have a beneficial effect on the maintenance of endothelial function. The development of structural atherosclerosis requires circulating CD34-positive cells [11]. Overweight, which is positively associated with fast eating [4], has also been reported to be positively associated with structural atherosclerosis specifically with respected to arterial wall thickness evaluated by carotid intima-media thickness [10]. Further studies with information about circulating CD34-positive cells is necessary to clarify those mechanisms.

Inflammation is a known contributor to both intervertebral disc degeneration [30] and osteoporosis [31], known risk factors for height loss. Since education level and economic status influence levels of inflammation [32, 33], education level and economic status could be associated with height loss. In addition, the prevalence of obesity is also reported to be influenced by education level and economic status [34]. In the present study, overweight was revealed to be positively associated with height loss. Therefore, education level and economic status could be underlying the associations between eating speed and height loss in relation to overweight status.

This is the first study to reveal the association between eating speed and height loss. Since height loss starting in middle age is a significant risk factor for cardiovascular diseases [3], the present findings could help clarify some of the mechanisms underlying the association between height loss and cardiovascular disease. Insufficient endothelial repair [7, 8], which is related to a shortage of circulating CD34-positive cells, is associated with height loss [6]. Circulating CD34-positive cell count is inversely associated with cardiovascular mortality [9]. Therefore, endothelial repair activity might play a critical role in causing height loss. However, measuring circulating CD34-positive cell count is not easy in daily clinical practice. The present study clarified that the risk of height loss might be helpful for assessing endothelial repair activity without measuring circulating CD34-positive cell count. Although further investigation with the data on endothelial repair activity is necessary, the present study suggests that eating speed might influence endothelial repair activity.

The clinical implication of the present study is that fast eaters who are not overweight have a high risk of height loss. Although fast eating was inversely associated with height loss among participants who were overweight, eating speed might be an important factor that influences BMI, which is also an independent risk factor for height loss. Then, even among overweight, slowing eating speed might be important to prevent height loss.

This study has some limitations. Intervertebral disc degeneration and vertebral fractures play important roles in height loss among adults. However, as in our previous study [5, 6, 14], we do not have data about those diseases. Most patients with intervertebral disc degeneration and vertebral fractures are asymptomatic [35, 36]. Therefore, plain radiographs, computed tomography, or magnetic resonance imaging are necessary to identify those diseases. In this study, the highest quintile of annual height loss was used to indicate height loss. However, an efficient cutoff point for defining height loss has not been established. Our additional analysis based on sextiles of annual height loss showed essentially the same associations. Although associations between eating speed and height loss were found to be independent of traditional cardiovascular risk factors in non-overweight individuals, other residual confounders such as education level and economic status might affect the associations. Eating speed was self-reported and was not objectively evaluated. However, evaluating eating speed by using a self-reported questionnaire has been shown to be valid [15, 16, 37]. Furthermore, self-reported eating rate has been shown to be correlated with objectively measured eating rate [38].

## 5. Conclusions

In conclusion, among Japanese workers who were not overweight, eating speed was positively associated with height loss; there was an inverse association among those who were overweight. However, fast eating was positively associated with overweight; overweight was positively associated with height loss. Since height loss starting in middle age is an independent risk factor for cardiovascular mortality, slowing eating speed might be beneficial for preventing height loss and cardiovascular disease.
