# Correction: Eating speed and height loss in relation to overweight: A retrospective study

**DOI:** 10.1371/journal.pone.0314218

**Published:** 2024-11-15

**Authors:** Yuji Shimizu, Hidenobu Hayakawa, Eiko Honda, Nagisa Sasaki, Midori Takada, Takeo Okada, Tetsuya Ohira, Masahiko Kiyama

The seventh author’s name is spelled incorrectly. The correct name is: Tetsuya Ohira.
